# Contribution of Endothelial Injury and Inflammation in Early Phase to Vein Graft Failure: The Causal Factors Impact on the Development of Intimal Hyperplasia in Murine Models

**DOI:** 10.1371/journal.pone.0098904

**Published:** 2014-06-02

**Authors:** Chi-Nan Tseng, Eva Karlöf, Ya-Ting Chang, Mariette Lengquist, Pierre Rotzius, Per-Olof Berggren, Ulf Hedin, Einar E. Eriksson

**Affiliations:** 1 Department of Molecular Medicine and Surgery, Karolinska University Hospital, Karolinska Institutet, Stockholm, Sweden; 2 The Rolf Luft Research Center for Diabetes and Endocrinology, Karolinska University Hospital, Karolinska Institutet, Stockholm, Sweden; Ludwig-Maximilians-Universität, Germany

## Abstract

**Objectives:**

Autologous veins are preferred conduits in by-pass surgery. However, long-term results are hampered by limited patency due to intimal hyperplasia. Although mechanisms involved in development of intimal hyperplasia have been established, the role of inflammatory processes is still unclear. Here, we studied leukocyte recruitment and intimal hyperplasia in inferior vena cava grafts transferred to abdominal aorta in mice.

**Methods and Results:**

Several microscopic techniques were used to study endothelium denudation and regeneration and leukocyte recruitment on endothelium. Scanning electron microscopy demonstrated denudation of vein graft endothelium 7 days post-transfer and complete endothelial regeneration by 28 days. Examination of vein grafts transferred to mice transgenic for green fluorescent protein under Tie2 promoter in endothelial cells showed regeneration of graft endothelium from the adjacent aorta. Intravital microscopy revealed recruitment of leukocytes in vein grafts at 7 days in wild type mice, which had tapered off by 28 days. At 28 and 63 days there was significant development of intimal hyperplasia. In contrast; no injury, leukocyte recruitment nor intimal hyperplasia occurred in arterial grafts. Leukocyte recruitment was reduced in vein grafts in mice deficient in E- and P-selectin. In parallel, intimal hyperplasia was reduced in vein grafts in mice deficient in E- and P-selectin and in wild type mice receiving P-selectin/E-selectin function-blocking antibodies.

**Conclusion:**

The results show that early phase endothelial injury and inflammation are crucial processes in intimal hyperplasia in murine vein grafts. The data implicate endothelial selectins as targets for intervention of vein graft disease.

## Introduction

Vein grafts (VGs) are preferred conduits for arterial reconstruction in patients with cardiovascular disease [1]. However, VGs display a limited patency with a rate of occlusion of approximately 10%–40% in one year, 30% in 5 years and 50% in 10 years after grafting [2], [3]. This is mainly due to intimal hyperplasia (IH). IH develops as early as 6 weeks after grafting in human VGs through proliferation and deposition of extracellular matrix by vascular smooth muscle cells (SMCs) that lead to narrowing of vessel lumen [4]. Whereas inflammation and leukocyte recruitment have been shown to be involved in this process in arterial injury models, the role of inflammatory processes in the formation of IH in VGs is largely unknown.

In contrast, arterial grafts (AGs) such as free radial artery grafts or in situ internal mammary artery grafts are less sensitive to IH and display better long-term patency rates [5]. The differences in IH between AGs and VGs implicate that IH has little to do with surgical trauma because both types of grafts are exposed to essentially the same treatment during cardiovascular procedures. Apparently, transfer of veins into a high pressure, high shear stress system triggers responses intrinsic in veins that ultimately lead to formation of IH [6], [7]. One possible difference between arterial and venous grafts may involve the endothelial phenotype. We have previously shown that the endothelium in large veins in mice possesses strong inflammatory capacity, which resembles that of the specialized inflammatory properties of post-capillary venules [8]. The introduction of such endothelium into the arterial circulation may thus trigger release of proinflammatory compounds, recruitment of leukocytes which may both influence development of IH. Here, we studied leukocyte recruitment and development of VG IH in C57BL/6 mice (WT mice) and in mice deficient in leukocyte adhesion molecules E- and P-selectin (EP^−/−^ mice). We show distinct early endothelial injury, leukocyte invasion and IH formation in VGs, but not in AGs. Furthermore, inflammation and IH was obviously reduced in EP^−/−^ mice. The data highlight the importance of selectin-dependent leukocyte recruitment in the development of IH in VGs.

## Materials and Methods

### Animals

C57BL/6 mice (WT mice) were obtained from B&K, Sweden and fed chow and water ad libitum. Tie2-GFP mice expressing green fluorescent protein (GFP) under Tie2 promoter [9] (Strain: STOCK Tg(TIE2GFP)287Sato/J) and appropriate control FVB mice (Strain: FVB/NJ) were obtained from Jackson labs (Bar Harbor, MN, USA) and were used to study the process of endothelial regeneration in VGs. EP-selectin double deficient mice (EP^−/−^ mice) were kindly provided by K. Ley and D.C Bullard and had been backcrossed into the C57BL/6 strain for at least 6 generations [10], [11]. EP^−/−^ mice were used to study leukocyte recruitment and IH development with the deficiency of E- and P-selectins. Experiments were approved by the regional ethical committee for animal experimentation (Karolinska institute Ethic permission number: N63/08, N103/11).

### Vascular grafting procedure

Transfer of the inferior vena cava (IVC) from donor mice into the abdominal aorta of recipients were performed by an end-to-end anastomosis according to a modified protocol [12]. Under isoflurane anesthesia, mice were heparinized with heparin 100 IU/kg body weight (KabiVitrum, Stockholm, Sweden) by intra-cardiac injection to the right atrium. The IVC between the right atrium and the diaphragm was subsequently harvested and the graft was placed in sterile medium (Medium-199, Sigma-Aldrich, Inc. St. Louis, MO, USA) at room temperature. Recipients were subsequently anesthetized, placed in a supine position, the abdomen opened by a midline incision, and the infra-renal abdominal aorta isolated from the adjacent IVC. Recipient mice were then heparinized by an intravenous injection through the left renal vein. The abdominal aorta was clamped with 2 micro-vascular clamps (size B-1; Fine Science Tools, Foster City, CA, USA) with a proximal clamp located below the renal arteries and a distal placed above the aortic bifurcation. The aorta was cut between the two clamps and interposed the VG with end-to-end anastomosis. As for AGs, the abdominal aorta in donors was harvested and transplanted in a similar procedure. Leakage in the anastomosis was carefully monitored after re-establishing blood flow.

Graft patency and blood flow was evaluated and the abdomen was closed in 2 layers. After surgery, mice were put in a 30°C chamber for recovery. Analgesia (Temgesic/Buprenorphinum, 0.05 mg/kg body weight, Schering-Plough Europe, Brussels, Belgium) was given subcutaneously every 6 hours during the first 24 hours and during the individual experiments.

Complementary experiments using control and antibody-treated WT mice were performed by cuff-assisted vein grafting in which VGs were transferred to the right common carotid artery [13]. This cuff-assisted vein grafting method is easier to use and develops similar IH as sewn technique in mouse and more suited to antibody treated WT mice [14]. The IVC was harvested and stored in sterile medium as previously described. With 2.5% Isoflurane anesthesia, recipient WT mouse was put in supine position and its neck was opened by a midline incision. The right carotid artery was isolated from adjacent tissue. The vessel was clamped proximally in the lower common carotid and distally across the carotid bifurcation. The vessel was cut and two cuffs were inserted into either arterial stump. The VG was subsequently placed between the two cuffed arterial stumps and ligated to either cuff. The clamps were then released to restore blood flow.

### Antibody injection

Rat anti-mouse P-Selectin antibody (Clone RB40.34) was kindly provided by Dr. Dietmar Vestweber in Max-Planck-Institute of molecular biomedicine, Muenster, Germany. Rat anti-mouse E-selectin antibody (Clone 9A9) was kindly provided by Dr. Klaus Ley in La Jolla Institute for Allergy & Immunology, San Diego, CA, USA and Dr. Barry Wolitzky. Rat IgG_1_ λ isotype immunoglobulin was obtained from BD Pharmingen (San Diego, CA, USA). Both Donor and recipient WT mice received the mixture of 100 µg antibody against P-Selectin and 100 µg antibody against E-selectin or 200 µg rat IgG_1_ λ isotype immunoglobulin intra-peritoneally 1 hour before vein grafting. These antibodies blocks functionally selectins for at least 7 days in an arterial injury model [15]. Antibodies were given every 5 days during a course of 28 days experimental period, with every mouse receiving a total of 6 injections.

### Graft harvesting

Grafts were harvested 5 min after removal of aortic clamp (Time 0), or at 7-, 28-, and 63 days following transfer. Deep anesthetic mice were perfused with 4% Zink formaldehyde (Histolab, Stockholm, Sweden) from the left ventricle puncture. VGs with the adjacent IVC were excised *en bloc* and immersed in 4% Zink formaldehyde. After dehydration in ethanol and xylene, grafts were embedded in paraffin and sectioned (5 µm) in a Zeiss HM 360 microtome (Carl Zeiss Meditec, Jena, Germany) and mounted on glass slides. Images were analyzed by ImageJ software (NIH, USA).

### Intravital microscopy

Under 2.5% isoflurane anesthesia, mice was put in a supine position, a catheter was placed in the left jugular vein and the abdomen opened with a midline incision and exposed tissue superfused by buffered saline at 37°C. The IVC and aorta were exposed inferior to the renal arteries by gentle dissection. Intravital microscopy was performed on the IVC, aorta or grafts between the renal and the iliac arteries. Blood samples (10 µL) were taken from the tail vein and analyzed for white blood count (WBC) in a Bürker chamber. Microscopic observations were made using a Leitz Biomed microscope with a Leitz SW25 water immersion objective. Epi-illumination fluorescence microscopy (Leitz Ploem-o-pac, filter block M2) was started 2 minutes after labeling of circulating leukocytes with an intravenous injection of Rhodamine 6G (0.67 mg/kg). Images were recorded on VCR using a VNC-703 CCD camera for offline analysis. Rolling leukocyte flux was determined as the number of leukocytes passing a 100 µm long reference line perpendicular to blood flow. Leukocyte adhesion was measured as the number of leukocytes firmly adherent for more than 30 seconds in a 100 µm×100 µm square of endothelium.

### Scanning electron microscopy (SEM)

Vessels were excised under anesthesia and immersed in 2.5% glutaraldehyde for 30 minutes, mounted *en face*, and dehydrated in increasing concentrations of ethanol, and CO_2_. After platina sputter coating, the vessels were examined in a ZEISS GEMINI Ultra 55 scanning electron microscope (Carl Zeiss Microscope, Jena, Germany). Images were analyzed by ImageJ software (NIH, USA).

### Confocal microscopy

Deep anesthetic Tie2-GFP or FVB mice were perfused through the left ventricle with phosphate-buffered 1.5% formaldehyde substituted with 10% glucose (100 mmHg, 20 min) with an outflow through the right atrium. Vessels were cut longitudinally and mounted in glycerol *en face* on glass slides, and examined in a Leica TCS SP5 confocal microscope (Carl Zeiss Microscope, Jena, Germany) at 490 nm and at 510 nm emission wavelengths using regular and oil immersion objectives (Leica Fluotar 20X/0.50 and 40X/1.25-0.15 OIL, respectively).

### Statistical analysis

The data represent mean±SEM of measurements obtained in indicated number of experiments. Statistical comparison between groups was performed using ANOVA with LSD post-hoc test. Statistical significance was set at p<0.05.

## Results

74 VGs (36 WT VGs, 14 FVB VGs, 6 Tie2-GFP VGs, 18 EP^−/−^ VGs) and 12 WT AGs were included for analysis. Grafted mice were carefully monitored and received postoperative analgesia. Overall success rates after an initial training stage were ∼50% in VG group and ∼80% in AG group. The causes of mortality were intraoperative bleeding, reperfusion injury and thrombus formation in grafts; where the two latter appeared to be dependent on aortic clamping time (on average VG: 66±1.7 vs. AG: 54±3.6 min).

### Denudation and regeneration of endothelium after grafting in WT mice

**Figure 1:**
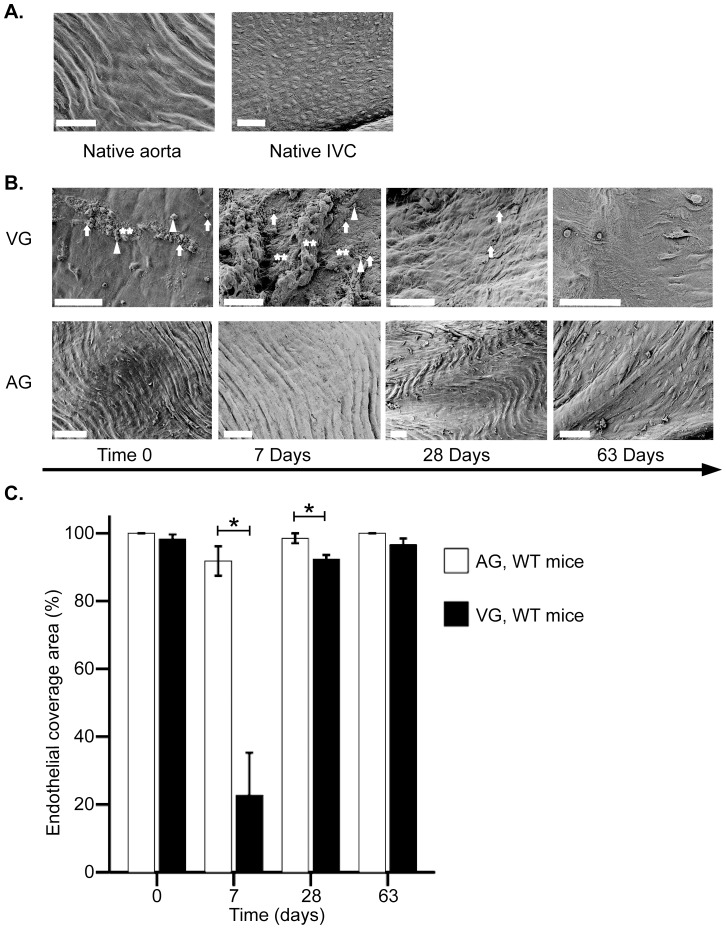
Endothelial structure in vascular grafts. Scanning electron microscopy images of endothelium in AGs and VGs (magnification 1K to 1.9K, scale bar = 50 µm). Images demonstrate endothelium in native aorta, native IVC (A), VGs and AGs (B) at different time point in WT mice. Bar graph shows endothelial coverage area change in AGs and VGs (C). Arrow = red blood cells, Triangle arrow = leukocytes, ** = denudated endothelium. Error bars represent mean±SEM. *p<0.05.

We used SEM to study VG endothelium after transfer of the IVC from donor mice to the abdominal aorta in recipients. Endothelium in native aorta and IVC showed confluent and no signs of injury under SEM (Figure 1A). However, directly after transfer (Time 0), the endothelium in VGs showed early signs of injury including partial denudation and platelet adhesion (98±1.3% endothelial cell coverage area; Figure 1B, C). At 7 days post-transfer, endothelial denudation in VGs was significant with a coverage area of only 23±13%, leaving sub-endothelial structures adhered to platelets, erythrocytes and leukocytes (Figure 1B). Endothelial coverage in VGs had largely recovered by 28 days (92±1.3%; Figure 1B, C). However, regenerated endothelium represented structurally irregular, uneven and with spindle-shaped individual cells (Figure 1B, C). In contrast to the finding in VGs, AGs did not show reduced endothelial integrity at any time point (Figure 1B, C) indicating that endothelial injury is specific for VGs.

**Figure 2:**
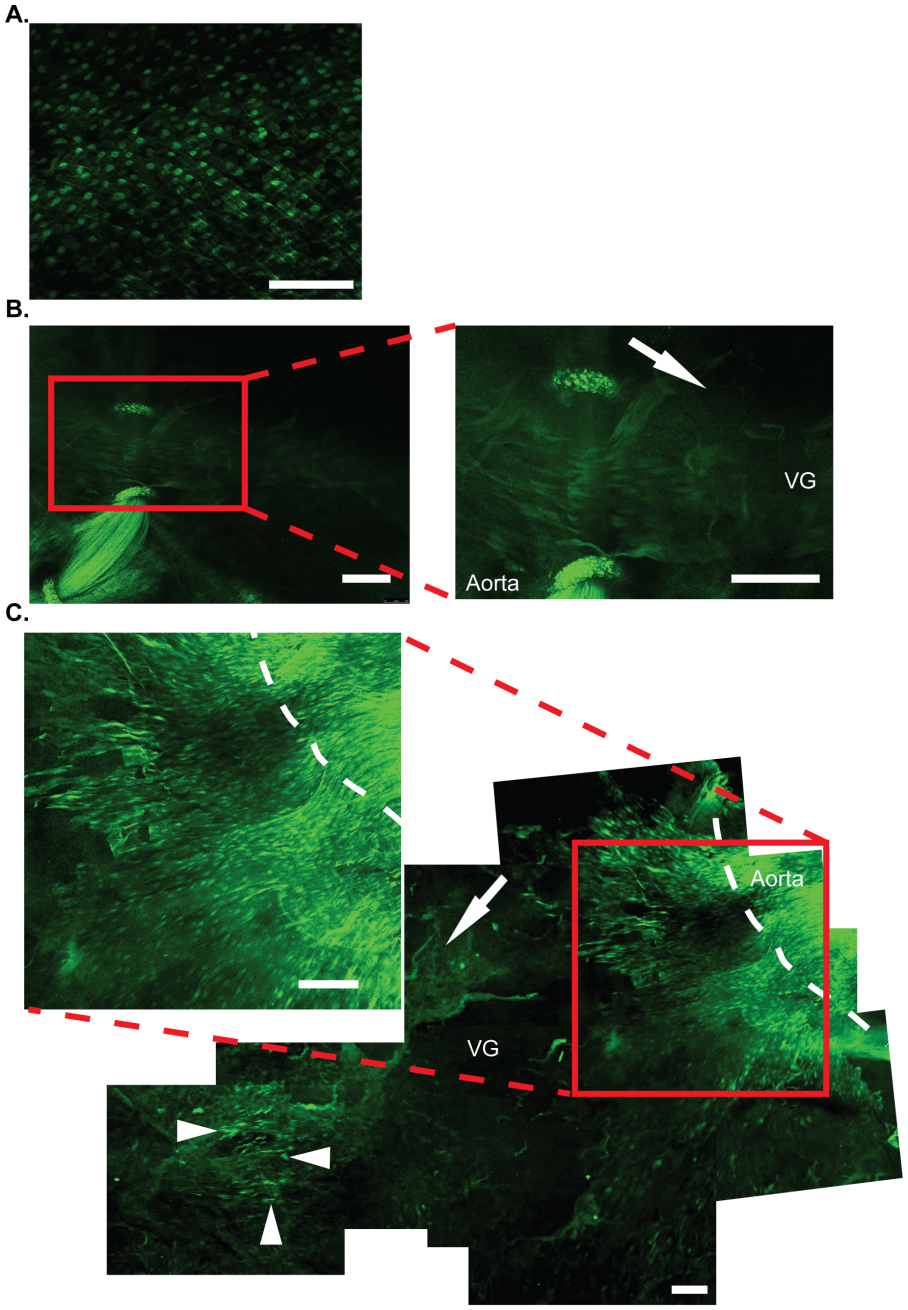
Endothelial regeneration in VGs. Confocal microscopy images of endothelium in IVC and in VGs. Endothelium of IVC in a Tie2-GFP mouse (A). Photomontage of confocal images of a FVB VG grafted into a Tie2-GFP recipient at 14 days (B) and at 28 days (C). The GFP-labeled endothelial cells located at center of a FVB VG at 28 days (C). Migration of GFP-labeled endothelial cells from aorta is evident by sequent time point. Zoomed images show photos in higher magnification. Isolated GFP-positive cells in center of grafts are also visible. Scale bar = 100 µm. Dotted line = Anastomosis between aorta and VG, Triangle head = isolated endothelial cells in middle of VG, Arrow = direction of blood flow.

In order to study neo-endothelium in VGs, we used confocal microscopy to investigate regeneration of fluorescent endothelial cells [16] from recipient Tie2-GFP mice into FVB VGs 14 days and 28 days after transfer. As shown previously, Tie2-GFP mice showed distinct GFP fluorescence in endothelial cells (Figure 2A). In Tie2-GFP recipient mice, GFP signals were detected primarily around anastomosis area. This finding demonstrated that endothelial cells were migrating from adjacent aorta (Figure 2B, C). Interestingly, GFP-positive endothelial cells were also observed in areas distant from anastomoses at 28 days (Figure 2C).

### Recruitment of leukocytes in VGs of EP^−/−^ and WT mice

**Figure 3:**
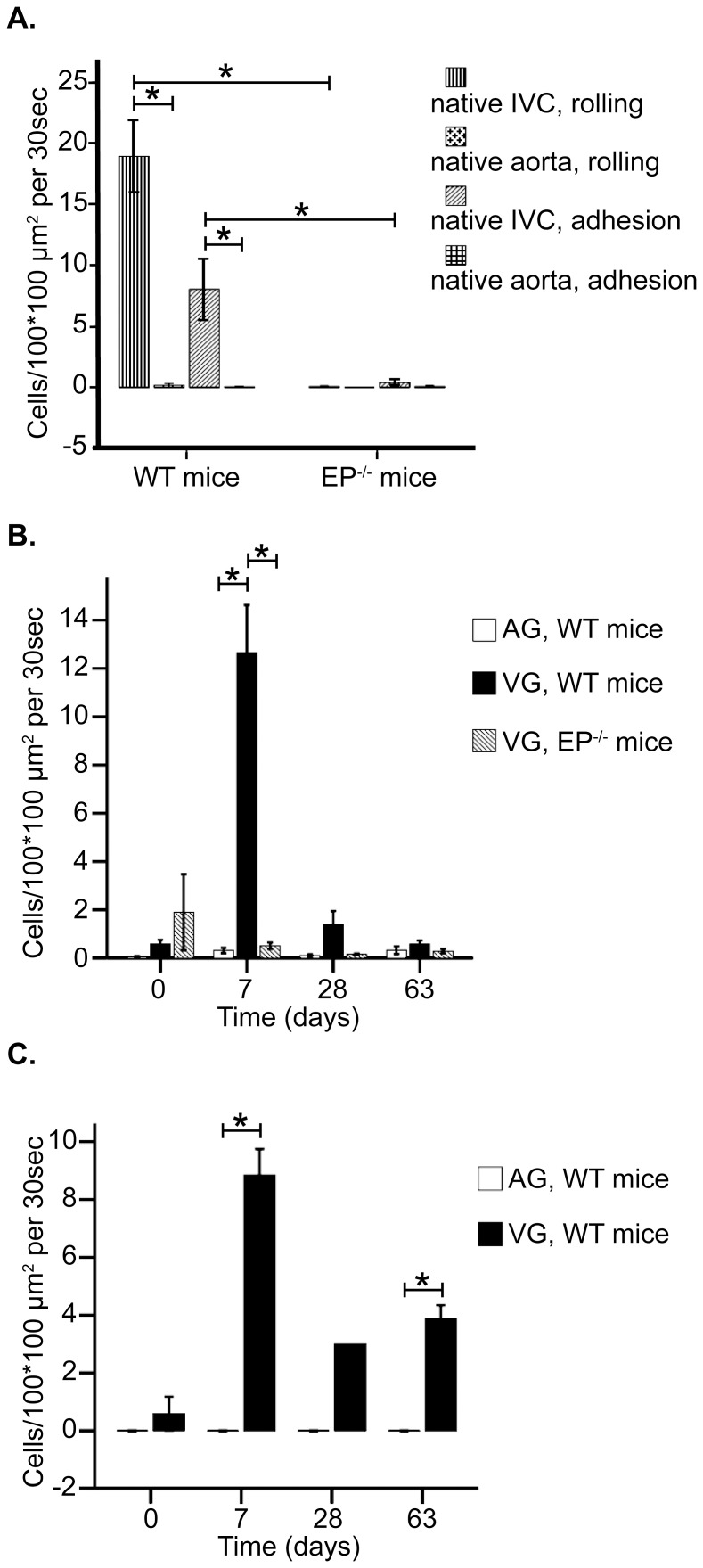
Leukocyte rolling and adhesion in native vessels and vascular grafts. Intravital microscopic data on leukocyte recruitment. Bar graphs represent (A) leukocyte rolling and adhesion in native vessels in WT and EP^−/−^mice, (B) leukocyte adhesion in AGs and VGs in WT and EP^−/−^ mice and (C) leukocyte rolling in AGs and VGs in WT mice at different time points. Bar graphs represent number of leukocytes that were visible in a 100×100 µm square during 30 seconds. Error bars represent mean±SEM. *p<0.05.

In order to investigate whether endothelium in VGs may promote inflammation and recruitment of leukocytes, we performed intravital microscopy on grafted mice. Here, baseline leukocyte adhesion in a 100 µm ×100 µm square in the native aorta and IVC in WT mice was 0.060±0.020 and 8.0±2.5 cells/30 sec, respectively (p<0.01; Figure 3A). Respective numbers of rolling leukocytes followed trend of adhesion in the native aorta and IVC in WT being 0.17±0.15 and 19±3.0 cells/30 sec, respectively (p<0.01; Figure 3A). As expected, the native IVC in EP^−/−^ mice showed reduced adherent as well as reduced rolling leukocytes compared to IVC in WT mice (0.39±0.26 adherent cells and 0.060±0.060 rolling cells/30 sec in EP^−/−^mice, p<0.01; Figure 3A).

After grafting, there was an apparent difference between VGs and AGs in leukocyte adhesion at 7 days (13±2.0 vs. 0.32±0.11 cells/30 sec in VGs and AGs, respectively, p<0.01; Figure 3B). However at 63 days, leukocyte adhesion in re-endothelialized VGs was similar to that in AGs (0.59±0.15 vs. 0.33±0.16 cells/30 sec in VGs and AGs; Figure 3B). In parallel, leukocyte rolling was abundant in VGs but not in AGs at 7 days post-transfer (8.5±0.62 vs. 0 cells/30 sec, p<0.01; Figure 3C). A similar difference in leukocyte rolling between VGs and AGs was also detected at 63 days (3.9±0.45 vs. 0 cells/30 sec in AGs, p<0.01; Figure 3C).

In order to investigate contribution of E- and P-selectin to leukocyte recruitment in VGs, we also studied EP^−/−^ mice grafted with EP^−/−^ VGs with intravital microscopy. At 7 days, significantly reduced numbers of leukocytes adhered to VGs endothelium (0.51±0.14 vs. in WT mice, p<0.01; Figure 3B), despite that the number of circulating leukocytes is massively increased in EP^−/−^ mice [17]. As mentioned above, leukocyte adhesion was reduced in WT mice at 63 days and there was no significant difference between EP^−/−^ mice and WT mice at this time point (0.29±0.090 vs. 0.59±0.15 cells/30 sec; Figure 3B).

### IH in VGs is reduced by inhibition of P- and E- selectin

**Figure 4:**
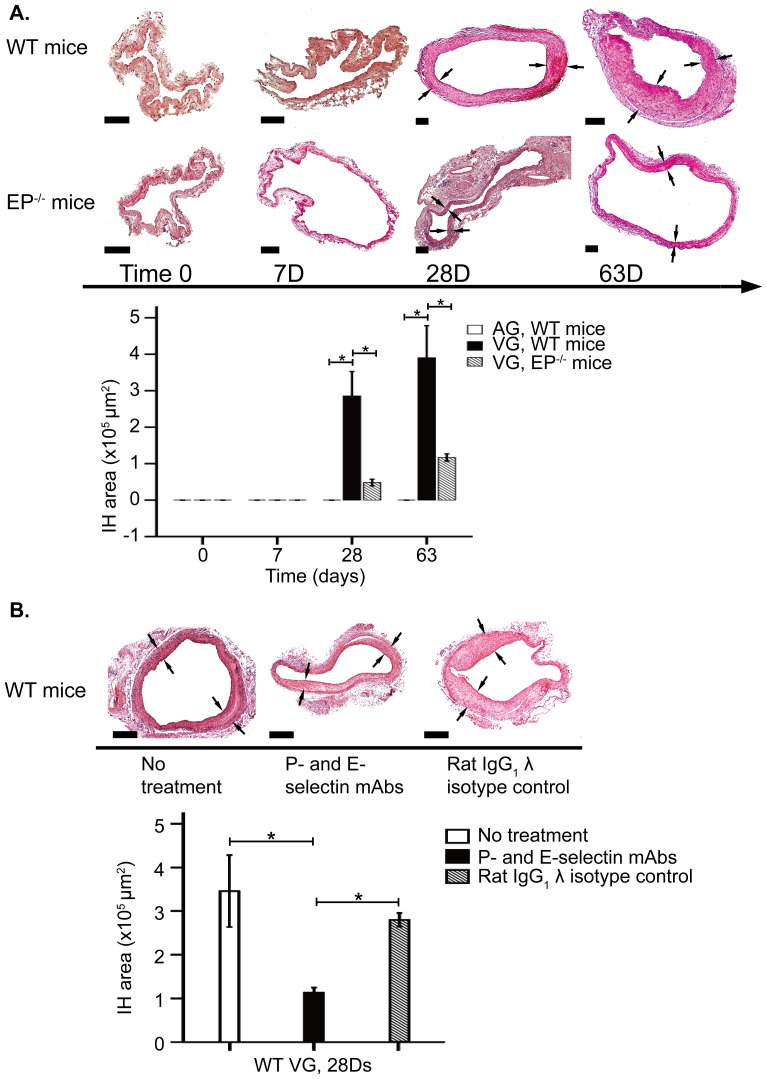
IH in vascular grafts. Morphologic assessment of vascular grafts stained with Hematoxylin-eosin. (A) Upper panel demonstrates VGs in WT mice and in EP^−/−^ mice at different time point. Bar graphs represent IH area in AGs and VGs from WT and VGs from EP^−/−^ mice. (B) Lower panel demonstrates VGs in WT mice without or with treatment with combination of function-blockage antibodies against P- and E-selectin or rat IgG_1_ λ isotype control in cuff-assisted vein grafting technique. Bar graph represents IH area. Error bars represent mean±SEM. *p<0.05. Scale bar = 100 µm. Arrows indicated IH of VGs.

In order to clarify if absence or function-blockage of leukocyte recruitment molecules P- and E-selectin could influence not only leukocyte recruitment but also IH development, we studied the development of IH in WT mice and EP^−/−^ mice and in WT mice treated with P- and E-selectin function-blocking antibodies. We found that VGs in WT mice developed significant IH by 28 days post transfer. In contrast, AGs did not display IH at the same time point (VGs 2.9±0.67×10^5^ vs. AGs 0 µm^2^, p<0.01; Figure 4A). No IH was found in AGs also at 63 days whereas IH development in VGs slowed down between 28 and 63 days compared to first 28 days (VGs 3.9±0.88×10^5^ vs. AGs 0 µm^2^, p<0.01; Figure 4A). Interestingly, VGs in EP^−/−^ mice developed less IH than WT mice both at 28 (0.48±0.090×10^5^ vs. 2.9±0.67×10^5^ µm^2^, p<0.05; Figure 4A) and 63 days (1.2±0.10×10^5^ vs. 3.9±0.88×10^5^ µm^2^, p<0.05; Figure 4A).

In order to confirm this finding, we used cuff-assisted vein grafting to carotid artery. In this model, IH in WT mice was also significant at 28 days (3.5±0.83×10^5^ µm^2^; Figure 4B). However, IH was strongly reduced in WT mice treated with a combination of function-blocking antibodies against P- and E-selectin (1.1±0.12×10^5^ µm^2^ vs. WT mice, p<0.05; Figure 4B) whereas there was no change in IH development in mice receiving an irrelevant isotype control (2.9±0.16×10^5^ µm^2^ vs. mixture of P- and E-selectin mAb treated WT mice, p<0.05; Figure 4B).

## Discussion

Surgical grafting of arteries and veins remains a principal strategy for treatment of organ ischemia secondary to arterial disease. However, IH limits patency and function in VGs. Several reports have elucidated roles of inflammatory molecules in IH progression in VGs [4], [18], [19]. These include cytokines such as TNFα [20], cell adhesion molecules like ICAM-1 [18], growth factors such as bFGF and PDGF [4], and vasoactive compounds like endothelin [21]. Experimental data thus encourages further studies of IH inhibition by interfering with similar molecular pathways in humans [22]–[24]; however intervention with these pathways has not reached clinical use. In this study, we investigate the role of leukocyte recruitment and inflammation on development of IH in two mouse models of vascular grafting. We demonstrate extensive endothelial injury in response to grafting which was specific for VGs and absent in AGs. Loss of endothelial integrity was associated with recruitment of leukocytes, which was attenuated in mice deficient in E-, and P-selectin despite massively increased numbers of circulating leukocytes in these mice. Furthermore, genetic deficiency or function-blockage of E- and P-selectin reduced IH in VGs. Hence, VG-specific inflammatory responses dependent on E- and P-selectin are crucial for IH development.

It has been hypothesized that arterial hemodynamics involving arterial pressure, vessel tension, pulsations, and shear stress may trigger inflammatory responses in VGs [4], [6]. In agreement with previous reports, the effects seen in this study involve injury to venous endothelium, which peaked 7 days after surgery [2], [25], alterations that were not seen in AGs. This demonstrates not only an expected tolerance of AGs to the arterial environment but also that endothelial injury in VGs is not coupled to surgical trauma imposed by graft harvesting or implantation. Thus, endothelial injury, leukocyte accumulation and IH are dependent on factors intrinsic in veins.

We have previously shown that large vein endothelium in contrast to that of large arteries responds strongly to inflammatory stimuli with expression for inflammatory molecules and recruitment of leukocytes to vessel wall, the latter which was reduced in mice deficient in P-selectin, E-selectin or both these molecules [8]. Hence, these distinct properties of AGs and VGs involving endothelial function and leukocyte accumulation may influence development of IH. Interestingly, in our experiments of vein grafting, endothelial injury and leukocyte accumulation in VGs were distinct only during the first days to weeks after grafting and were returned almost to normal by 28 days following surgery. However, selectin-dependent interactions between leukocytes and the vessel wall were more prominent 7 days after grafting compared to 28 days. This may indicate that platelet-leukocyte interactions were involved in leukocyte adhesion to the vessel wall, as has previously been described in a reports showing importance of platelet P-selectin in development of IH in a wire injury model in mouse carotid arteries [26]. However, it is also possible that the remaining endothelium at 7 days is strongly activated and thus expressing P-selectin and inducing rolling and recruitment of leukocytes. Regardless, the data suggest that the structural and inflammatory damage suffered by VGs is of importance mainly at early stages indicating some adjustment of VGs to arterial hemodynamic conditions after an initial phase of adaptation. It is tempting to suggest that this adjustment involves IH development in itself since IH will strengthen the vessel wall and influence vascular tension and possibly also endothelial stability.

Moreover, regeneration of injured graft endothelium by migrating aortic endothelial cells or circulating endothelial progenitor cells with phenotypes different to that of the grafted vein may also influence these parameters. It has been shown previously that inflammatory activity can enhance endothelial regeneration [27]–[29]. However, inhibition of leukocyte recruitment in mice deficient in E- and P-selectin had no obvious effect on endothelial regeneration in our model. Regardless, the reduced recruitment of leukocytes in early stages and later attenuated IH in mice with inhibited function of E- and P-selectin suggest that these molecules may be of importance during the first weeks after revascularization [18]. In parallel, Phillips et al. shows IH is reduced by 50–80% at 28 days after a single dose of P-selectin antibody in conjunction with a wire injury procedure in mouse carotid artery [15]. Together, these results indicate that early inhibition of selectins may present an attractive treatment strategy against IH both in arteries and in VGs; however further work is required to investigate potential for short-term inhibition of inflammation in VGs.

In conclusion, we demonstrate early inflammatory changes in veins grafted to the arterial circulation in mice, which are linked to subsequent development of IH. Further studies of inflammation in VGs and identification of key target processes that are distinct between veins and arteries may present treatment options to improve patency of VGs in patients after bypass surgery.
